# Draft Genome Sequence of the UV-Resistant Antarctic Bacterium *Sphingomonas* sp. Strain UV9

**DOI:** 10.1128/MRA.01651-18

**Published:** 2019-02-14

**Authors:** Juan J. Marizcurrena, Danilo Morales, Pablo Smircich, Susana Castro-Sowinski

**Affiliations:** a Section Biochemistry, Faculty of Sciences, Universidad de la República, Iguá, Montevideo, Uruguay; b Laboratory of Molecular Interactions, Faculty of Sciences, Universidad de la República, Iguá, Montevideo, Uruguay; c Department of Genomics, Instituto de Investigaciones Biológicas Clemente Estable, MEC, Montevideo, Uruguay; d Molecular Microbiology, Instituto de Investigaciones Biológicas Clemente Estable, MEC, Montevideo, Uruguay; Queens College

## Abstract

We report the draft genome sequence of the Antarctic UV-resistant bacterium *Sphingomonas* sp. strain UV9. The strain has a genome size of 4.25 Mb, a 65.62% GC content, and 3,879 protein-coding sequences. Among others, genes encoding the resolving of the DNA damage produced by the UV irradiation were identified.

## Abstract (precis)

We report the draft genome sequence of the Antarctic UV-resistant bacterium *Sphingomonas* sp. strain UV9. The strain has a genome size of 4.25 Mb, a 65.62% GC content, and 3,879 protein-coding sequences.

## ANNOUNCEMENT

Bacteria from the genus *Sphingomonas* are *Alphaproteobacteria* (family *Sphingomonadaceae*) with 127 described species. They are found in a broad range of environments, such as soils, fresh and marine waters, and plants, and in humans acting as opportunistic pathogens (1–6). *Sphingomonas* strains also colonize extreme environments, including Antarctica, volcano lakes, contaminated soils, and highly UV-irradiated places (6–8). They are Gram-negative, rod-shaped, chemoheterotrophic, strictly aerobic, and non-spore-forming bacteria (9).

This work reports the draft genome sequence of the UV-resistant bacterium *Sphingomonas* sp. strain UV9. The isolation and growth conditions for UV9 were previously described (7). Total DNA was extracted using the fungal/bacterial DNA miniprep purification kit (Zymo Research, catalog number D6005). The library preparation was performed using the Accel-NGS 2S PCR-free DNA library kit (Swift Biosciences, MI) and was sequenced at Macrogen using the HiSeq 2000/2500 technology platform with 101-bp paired-end read strategy. At least 13.7 million reads were obtained. Their quality was evaluated with FastQC (https://www.bioinformatics.babraham.ac.uk/projects/fastqc/) and assembled *de novo* using SPAdes (http://cab.spbu.ru/software/spades/) with the repeat resolution and mismatch correction settings enabled. The draft genome consists of ca. 4.25 Mb, including 62 contigs of above 1,000 bp, with a GC content of 65.62% and an *N*_50_ contig length of 1.26 Mb (*L*_50_ of 2 Mb) and 40× final coverage. The genome was annotated and the functions of genes were predicted and compared using the Rapid Annotations using Subsystems Technology (RAST) (10) and NCBI Prokaryotic Genome Annotation Pipeline (PGAP) servers. The predicted genes were functionally categorized using the SEED subsystems (11) at the RAST server. Proteins that conserve functional domains were identified using the NCBI conserved domain search service (CD-Search) (12).

The genome was predicted to have at least 3,879 protein-coding sequences (CDS) (1,274 were considered hypothetical, and 1,750 CDSs were classified into 209 subsystems), 50 tRNAs, 1 copy each of 23S rRNA-, 16S rRNA-, and 5S rRNA-encoding genes, and 86 pseudogenes. UV9 has the genomic information for the production of three photolyases, enzymes responsible for photorepairing the DNA damage caused by UV irradiation (13). These include two photolyases that repair the cyclobutane pyrimidine dimers (CPD-photolyase) and one that repairs 6,4 photoproducts (6,4-photolyase); both photoproducts halt RNA polymerase II during transcription or DNA polymerase during replication (14). These enzymes may have different functional antenna chromophores, 8-hydroxy-7,8-didemethyl-5-deazariboflavin (MTHF) and/or 6,7-dimethyl-8-ribityllumazine (DMRL), as the biosynthetic pathways were found. UV9 also shows a UvrABC system (14) (excinuclease ABC), as is found in the gamma radiation-resistant Hymenobacter sedentarius (15) and Deinococcus swuensis (16) bacteria, and an ATP-dependent DNA helicase UvrD/PcrA (essential during replication, recombination, and repair of UV damage) (17). It also contains a copy of the *radA* gene (which fills a gap using the information from the undamaged DNA strand) and the DNA mismatch repair proteins MutL/MutS (which identify and correct errors made during the replication). UV9 has genetic information for the synthesis of bacteriorhodopsin, a light-driven proton pump. Finally, UV9 harbors heavy metal resistance genes, including those for cobalt, zinc, cadmium, chromium, and arsenic, and employs the toxin-antitoxin system (RelEB and VapC), including specific proteases such as Lon, ClpXP, or ClpAP that commonly degrade the antidote (18). Thus, strain UV9 could be a model for studying bacterial UV resistance.

### Data availability.

This whole-genome shotgun project has been deposited at DDBJ/ENA/GenBank under the accession number SCIN00000000. The version described in this paper is version SCIN01000000.
